# The Interaction Effects of Burnout and Job Support on Peptic Ulcer Disease (PUD) among Firefighters and Policemen

**DOI:** 10.3390/ijerph16132369

**Published:** 2019-07-03

**Authors:** Ping-Yi Lin, Jong-Yi Wang, Dann-Pyng Shih, Hsien-Wen Kuo, Wen-Miin Liang

**Affiliations:** 1 Transplant Medicine and Surgery Research Centre, Changhua Christian Hospital, Changhua 50006, Taiwan; 2 Department of Medical Research, China Medical University Hospital, Taichung 40402, Taiwan; 3 Department of Nursing, Da-Yeh University, Changhua 51591, Taiwan; 4 Department of Health Services Administration, China Medical University, Taichung 40402, Taiwan; 5 International Medical Department, Changhua Christian Hospital, Changhua 50006, Taiwan; 6 Department of Public Health, China Medical University, Taichung 40402, Taiwan; 7 Institute of Environmental and Occupational Health Sciences, National Yang Ming University, Taipei 11221, Taiwan; 8 School of Public Health, National Defense Medical Center, Taipei 11490, Taiwan

**Keywords:** burnout, job support, peptic ulcer diseases (PUD), firefighters, policemen

## Abstract

Policemen and firefighters encounter numerous emergency events that frequently lead to high burnout and low job support, resulting in adverse health effects. A number of studies reported the correlation between job characteristics and the risk of peptic ulcer diseases (PUD) across various industries. However, there is very little research on evaluating the interaction effects of burnout and job support on the prevalence of PUD among firefighters and policemen. The objective of this study was to assess the interactional effects between burnout and job support on the prevalence of PUD among firefighters and policemen. This was a cross-sectional quantitative study. Registered, full-time police officers and firefighters in Taiwan were anonymously interviewed by a mail-delivered questionnaire. All female workers were excluded due to different job characteristics and a limited sample size. A total of 9328 firefighters and 42,798 policemen completed the questionnaire, with a response rate of 78.7%. Overall, prevalence rates of self-reported and self-reports of physician-diagnosed PUD were 8.3% and 6.5% for policemen and 7.1% and 5.5% for firefighters, respectively. There was a 22% reduced odds ratio of PUD as diagnosed by physicians for the group with low burnout and high job support, but an increased odds ratio of 53% for the group with high burnout and low job support, after adjusting for lifestyle and demographic variables. There must be an increase of job support and reduction of burnout through the modification of work structure and setting up of counseling services to reduce workplace stress and the prevalence of PUD among policemen and firefighters.

## 1. Introduction

Policemen and firefighters frequently encounter emergencies at a higher rate than other public service workers, which leads to burnout and affects their physical and mental health. Peptic ulcer diseases (PUD) are significantly related to long work shifts (odds ratio, OR: 2.46; 95% confidence interval, CI: 1.06–5.73) and with occupational stress (OR: 2.28; 95% CI: 1.16–4.49) [1]. PUD are the ulcers that affect both the stomach and small intestines, which allows the digestive acids to eat away at the tissues that line the stomach. Stomach ulcers are mostly caused by an infection with the bacterium *Helicobacter pylori* (*H. pylori*) or long-term use of nonsteroidal anti-inflammatory drugs (NSAIDs), such as aspirin, ibuprofen, or naproxen. Some of the more common symptoms may include abdominal pain, burning, nausea, bloating, or black stools. The self-reported PUD prevalence among people aged 18 years and over in the United States was 10.3% in 1989 [2]. A study in Taiwan indicated a PUD prevalence rate of 9.4% in asymptomatic subjects who had low education, had a high body mass index (BMI), and were current smokers [3]. A stressful life is not usually included in the list of stomach ulcer causes, but once the PUD are present, stress can become the major cause for worsening the condition. Psychological stress can contribute to the development of PUD and can exacerbate the symptoms of existing conditions, making stress a probable cofactor with *H. pylori*. Psychological stress most likely interacts with *H. pylori* and other risk factors in causing PUD. There are also positive interaction effects between perceived job stress and smoking on the development of PUD [4].

A study in Taiwan showed nurses and other health care workers having a significantly higher PUD risk than the general population (OR: 1.48; 95% CI: 1.43–1.52 and OR: 1.33; 95% CI: 1.25–1.42, respectively), but pharmacists had a lower risk (OR: 0.88; 95% CI: 0.83–0.95) and physicians had a non-significant difference in risk [5]. Health care workers have similar job characteristics as policemen and firefighters, such as stressful workloads, frequent shift changes, overnight work, and erratic work schedule. In addition, policemen and firefighters have frequent exposure to crises and high-pressure situations [6,7,8]. Consequently, the “stress reactions” may release more stress hormones (norepinephrine and serotonin) to initiate adaptive mechanisms, as well as counteract the stress-induced gastric mucosal damage [9]. Psychological stress may affect gastric secretion, gut motility, mucosal permeability, mucosal barrier function, visceral sensitivity, and mucosal blood flow [9]. Policemen and firefighters experience high work-related stress and burnout. A number of studies reported the correlation between job characteristics and the risk of PUD across various industries [10,11]. However, there is very little research on evaluating the interaction effects of burnout and job support on the prevalence of PUD among firefighters and policemen. The objective of the study was to assess the interaction effect of burnout and job support on the prevalence of PUD among firefighters and policemen.

## 2. Materials and Methods

### 2.1. Study Population

This was a cross-sectional quantitative study. Data was obtained from Taiwan’s Health Promotion Administration (HPA). All full-time policemen and firefighters nationwide were invited to participate in the study by anonymously filling out a mail-delivered questionnaire. A total of 81,754 questionnaires were mailed to 89 institutions and the response rate was 79.7%. After removing the invalid questionnaires, the total number of questionnaires received was 64,371, resulting in a response rate of 78.7%. The reasons for non-response included vacations, requested time off, or having limited time to fill out the questionnaire, and they did not affect the objective of the study. This study excluded the questionnaires from the environmental protection staff and female workers due to different job characteristics and a limited sample size. A total of 52,126 participants were included in the analysis of the study. The study had ethical approval from the China Medical University (CMUH105-REC3-091).

### 2.2. Exposure and Outcome Measures

The questionnaire included demographic data, job characteristics, history of diseases, burnout levels, workload levels, and job support. The occupational burnout tool was obtained from [12], which was used as a screening tool in the workplace to identify high-tasks workers. Burnout scale included 8 items for personal and work-related burnout that were measured with a Likert’s scale: always (6 points), frequently (5 points), sometimes (4 points), occasionally (3 points), seldom (2 points), and none (1 point). The maximum score was 32, and a higher score represented a higher burnout level. The workload scale included five items: work pressure, exhaustion, heavy workload, limited time, and poorly defined tasks. Job support was measured by 8 items, which included work support and guidance by colleagues and superiors. A Likert’s scale was used to measure workload and job support: strongly agree (4 points), agree (3 points), disagree (2 points), and strongly disagree (1 points). PUD included stomach ulcers and intestinal ulcers diagnosed by physicians or reported by participants. Participants with PUD frequently reported symptoms of abdominal pain, burning, nausea, bloating, and black stools. Self-reported PUD’s were not confirmed by standard gastroscopy test, but symptoms of PUD can be classified as initial or moderate levels of PUD. The physicians diagnosed PUD using gastroscopy and *H. pylori* was searched by biopsy. Participants reported have been diagnosed with PUD by physician currently were defined as self-reports of physician-diagnosed PUD group.

### 2.3. Statistical Analysis

SPSS 24 package (IBM Corp, Armonk, NY, USA) was used to analyze the data. Chi-squared was used to test the correlation between demographic data and job characteristics for participants diagnosed with PUD by both themselves and physicians. Prevalence rates of burnout, workload, job support and peptic ulcers were compared using the Chi-square test between firefighters and policemen. Two models were used to indicate the correlation of burnout, workload, and job support with PUD diagnosed by both physicians and themselves using multiple logistic regression. A crude model that did not adjust for covariates was performed first. Then, a second model adjusted for groups, educational level, marital status, duration of job, levels of employing government, manager, smoking, alcohol, and betel nut chewing was conducted. Interaction effects of burnout and job support on the prevalence of PUD self-reported diagnosed by physicians and reported by participants themselves were assessed using multiple logistic regression models. Interaction between burnout and job support was assessed using two multiple logistic regression models adjusted for covariates. A *p*-value (two tailed) < 0.05 was considered statistically significant. 

## 3. Results

### 3.1. Descriptive Information of PUD

Table 1 shows the correlation between demographic information and peptic ulcers diagnosed by physicians and reported by participants. The prevalence of self-reported PUD was higher than of those diagnosed by physicians. There are significant differences in all demographic variables. The prevalence of PUD among the policemen and firefighters increased with age. The highest prevalence of PUD was found among subjects with senior high school education level. Divorced and widowed subjects had higher prevalence of PUD, and it occurred among self-diagnosed PUD subjects 1.5-fold as often as among self-reports of physician-diagnosed ones. Subjects who reported smoking, alcohol consumption, and betel nut chewing had a significantly higher prevalence of PUD. Occasional and frequent drinkers had a higher prevalence of PUD than nondrinkers.

Table 2 indicates the correlation between job characteristics and PUD among self-reported and physicians-diagnosed respondents. Prevalence rates of self-reported and self-reports of physician-diagnosed PUD in policemen (8.3% and 6.5%, respectively) were significantly higher than those in firefighters (7.1% and 5.5%, respectively). Persons with long work duration and superior position had a higher prevalence of PUD, but participants with overnight work had none. There is a significant difference in PUD prevalence among both on-call workers and those having different work schedules. Whether at home or in the office, both on-call groups had a greater prevalence of PUD. 

### 3.2. Job Characteristics and PUD

Table 3 compares the percentage of burnout, high workload, job support, and PUD among firefighters and policemen. The cut-off points for high burnout, high workload, and high job support were used on the 75% percentage excess of the scores. Except for job support, there are significant differences for burnout, workload, and PUD rates in both self-reported diagnosed by physicians and self-reported by themselves in policemen and firefighters. The percentages of high burnout and high workload among firefighters were 24.2% and 45.2%, which was higher than those among the policemen with 22.5% and 28.6%. However, self-reports of PUD diagnosed by physicians (6.5%) and self-reported (8.3%) by policemen were significantly higher than those among firefighters (5.5% and 7.1%, respectively).

### 3.3. Associated Factors of PUD

Table 4 shows self-reported and self-reports of physician-diagnosed PUD correlated with burnout, workload, and job support using multiple logistic regression. In the crude model, there was a positive correlation between burnout and workload with the prevalence of PUD. In the adjusted model, participants with high burnout and high workload had the higher prevalence of PUD after adjusting for smoking, alcohol and betel nut chewing. However, participants with high job support had lower prevalence of PUD in the adjusted models. This result is consistent with the reduced odds ratio of contracting PUD by 27–40% in the group enjoying high job support.

### 3.4. Interaction Effects on the Prevalence of PUD

Table 5 indicates the interaction effects between burnout and job support in relation to developing odds ratio of PUD, using multiple logistic regression models. Burnout and job support were classified into four levels (low burnout and low job support, low burnout and high job support, high burnout and low job support, high burnout and high job support). Based on the crude model, higher odds ratio of PUD in the high burnout and low job support group were 1.64 and 1.34, respectively, but a significantly lower odds ratio occurred in the group with low burnout and high job support, indicating PUD odds ratio reduction by 37% and 26%. Multiple logistic regression adjusted for covariates was used, resulting in findings consistent with those in Model 1. The groups with low burnout and high job support experienced the reduction of PUD odds ratio by 33% and 22% compared to the group with low burnout and low job support. However, there was an increase of 78% and 53% in the groups with high burnout and low job support.

## 4. Discussion

The prevalence rates of self-reported and self-reports of physician-diagnosed PUD were 8.3% and 6.5%, for policemen and 7.1% and 5.5% for firefighters, respectively. There was a significantly higher prevalence rate of PUD for policemen. Compared to health care workers (HCW) in Taiwan, the prevalence of PUD in firefighters and policemen, as diagnosed by physicians, was considerably lower than physicians (10.3%), nurses (9.5%), pharmacists (9.1%), and other HCWs (9.1%) [5]. In Japan, the lifetime risk of PUD in male workers was estimated at 23%, with an incidence rate of 5% or more per year [13]. A systematic review article reported greater differences in the worldwide incidence rate of PUD. The findings showed the highest annual incidence rates of all PUD and bleeding PUD were 141.8 per 100,000 persons in Spain and 72.5 per 100,000 persons in Greece, and the lowest was 8.3 per 100,000 persons in the UK. In addition, the highest annual incidence of perforated PUD was 4.4 per 100,000 persons in South Korea, and the lowest was 2.2 per 100,000 persons in the UK [14]. Factors influencing incidence rates of PUD can include cultural, diet, and lifestyle differences. Worldwide, the main cause of PUD remains *H. pylori* infection [15]. However, in some contexts, an increasing number of unexplained PUD is reported. In agreement with this latter event, even though there was a sharp decrease in *H. pylori* infections, the prevalence rate of PUD did not decrease as much in the same time period. In a nationwide twin cohort study [16], there was a 33% risk of PUD being attributed by genetic factors and 61% by environmental factors. In addition, there was a higher risk by 2.2 times and 3.2 times in men attributed to current smoking and having high stress levels. Similarly, the group with higher stress levels had a 2.2-fold risk of PUD (hazard ratio, HR 2.24; CI 95% 1.16:4.35), using the Cox proportional hazard regression after adjusting for covariates [17]. Although the etiology of human PUD is still unclear, *H. pylori* cannot serve as the sole explanation for PUD. A Chinese study [18] found a weak association between the prevalence of PUD, mainly duodenal ulcers, and the decreasing trend of the prevalence of *H. pylori* infection and NSAIDs. Therefore, the associated factors of PUD are possibly attributed to individual lifestyle differences and stressful work characteristics. The accumulated evidence suggests a complex multifactorial pathogenesis and regional variation of 5%–10% for the prevalence of PUD.

Policemen and firefighters frequently engage in stressful circumstances that can affect their psychological state. Due to the dramatic changes in the working environment in Taiwan, policemen and firefighters have been forced to work harder because of ongoing workplace restructuring [19]. They have suffered from psychological stress from their work, causing them to retire early or be officially acknowledged as victims of psychosomatic symptoms due to burnout. In addition, stressful working conditions also enhance the risk of adverse health effects or contribute to adopting behavior changes, such as smoking and alcohol consumption [20]. Workplace stress can also be caused by a poor work structure or organization, inflexible management system, and unsatisfactory working conditions, resulting in loss of control over their jobs and little support from the departments or organizations among policemen and firefighters [21]. A perspective intervention study [22] in different human service sector organizations found that psychosocial work characteristics were positively associated with work-related burnout, such as low possibilities for promotion, low sense of meaning in work, low role clarity, high pressure from leadership, and high role conflict. In addition, sickness absence for groups in the highest work burnout quartile was 13.9 days higher than the 6.0 days for groups in the lowest quartile [23]. A study in Chinese male off-shore workers with ulcer-like symptoms were positively associated with occupational stress and positively associated with the interaction effect between occupational stress and internal behavior coping [24]. However, they did not assess the correlation between job support and burnout with adverse health effects. Our findings indicated an interaction effect between burnout and job support on the prevalence of developing PUD. The prevalence of self-reported and physician- diagnosed PUD were reduced by 33% and 22% for the groups with low burnout and high job support, compared to those with low burnout and low job support. However, the prevalence of PUD increased by 78% and 53%, respectively, for the group with high burnout and low job support. In this study, evaluation of burnout in policemen and firefighters included huge workloads, long working hours, increasing job demands, feelings of frustration and exhaustion, and limited time to accompany family. Persons with long-term stress in an organization were more likely to develop burnout, thus causing lower performance and productivity and a higher risk of adverse health effects [25,26]. 

Our study findings were similar to [12], with the burnout levels positively correlated with job demands (r = 0.49 for men and women), perception of job stress (r = 0.50 for men and 0.53 for women), and levels of psychological distress (r = 0.68 for men and 0.65 for women). In addition, burnout levels were negatively associated with self-rated health status (r = −0.36 for men and r = −0.38 for women) and job satisfaction (r = −0.40 for men and r = −0.45 for women). The operational nature of policemen and firefighter jobs also include long shifts and overnight shifts, which frequently affect diet intake and psychological state. The nature of this work can disrupt the circadian rhythm and increase the odds ratio of ulcerogenic *H. pylori* infections. A study indicated that 34.6% of the shift workers and 16% of daytime workers had *H. pylori* positive infections but could not confirm a causal relationship between shift work and the development of PUD due to *H. pylori* infections [27]. Policemen and firefighters not only experienced burnout and workload stress, they frequently had unhealthy lifestyle habits and poor coping skills, which were significantly associated with the occurrence of PUD [28]. Cumulative exposure to environmental nighttime noise was contributed to the development of peptic ulcers (HR = 1.12; 95% Cl = 1.10–1.13) and gastric ulcers (1.17; 95% CI = 1.15–1.20) [29]. Consequently, a counseling system and training courses in dealing with occupational stress are needed to increase job support and modify job characteristics to reduce the prevalence of PUD in policemen and firefighters [30]. Also, it is necessary to prevent PUD by controlling environmental factors that predispose *H. Pylori* infections, such as having a balanced diet, exercising regularly, avoiding smoking, limiting alcohol intake, and getting sufficient sleep [28]. Hence, it is essential to increase the health literacy for public servants in order to eliminate the unhealthy behaviors, such as smoking, excessive drinking, and betel nut chewing.

This is the first nation-wide study that investigates the correlation between job characteristics and the prevalence of PU for policemen and firefighters. Our findings show the interaction effects between burnout and job support on the prevalence of PUD. However, several limitations exist in the study. Because the study was a cross-sectional design, it is hard to establish a causality relationship between job characteristics and the prevalence of PUD. In addition, our data did not confirm that each participant who suffered from PUD diseases was examined by standard gastroscopies. However, the national health insurance program in Taiwan provides accessible and comprehensive services for Taiwanese people, and a diagnosis of PUD by a physician must be based on a gastroscopy and an examination for the infection of *H. pylori*. Participants with self-reported PUD reported experiencing burning stomach pain, feeling of fullness, bloating or belching, fatty food intolerance, heartburn, or nausea. The pain was relieved by eating certain foods that buffer stomach acid or by taking an acid-reducing medication, but individuals might still have needed to go to the hospital. Finally, our findings may underreport the prevalence of PUD due to workers who retired early or could not fill out the questionnaire, which can underestimate the correlation between job characteristics and the prevalence of PUD. Meanwhile, this study also did not consider genetic heterogeneity, the use of drugs, such as NSAIDs, or personality type in contributing to the prevalence of PUD [31,32]. The combination of personality type and emotional stress also has a contribution to physiological functions in the gastrointestinal tract. It would be beneficial to implement an effective stress management program for public servants to reduce job-related psychological stress that induces gastrointestinal tract alterations [30].

## 5. Conclusions

The overall prevalence rates of PUD by self-report and diagnosis by physicians were 8.3% and 6.5% for policemen and 7.1% and 5.5% for firefighters, respectively. The interaction effect between burnout and job support was correlated with the prevalence of PUD. High job support and low burnout can reduce the prevalence of PUD for firefighters and policemen. However, low job support and high burnout can increase the prevalence of PUD. It is necessary to modify work structures and provide a counseling system to improve coping skills and strengthen job support to reduce the prevalence of PUD among policemen and firefighters.

## Figures and Tables

**Table 1:** Demographic information by self-reported and self-reports of physician-diagnosed peptic ulcer disease (PUD).

Demographic Information	Self-Report PUD *n* = 4205	*p*	Self-Reports of Physician-Diagnosed PUD *n* = 3309	*p*
Age (years)		<0.001		<0.001
20–29	307 (3.3%)		200 (2.1%)	
30–44	1767 (7.7%)		1377 (6.0%)	
45–54	1948 (10.9%)		1573 (8.8%)	
>55	168 (9.5%)		156 (8.8%)	
Education level		<0.001		<0.001
Senior high school	831 (10.8%)		600 (7.8%)	
College	2162 (8.1%)		1718 (6.4%)	
Undergraduate	958 (6.8%)		76 (5.4%)	
Graduate	199 (7.4%)		19 (7.1%)	
Marital status		<0.001		<0.001
Unmarried	527 (3.9%)		367 (2.7%)	
Married	3342 (9.4%)		2702 (7.6%)	
Divorce	258 (13.0%)		181 (9.1%)	
Widow	27 (13.8%)		18 (9.2%)	
Smoking habit		<0.001		<0.001
No	2546 (6.9%)		2096 (5.6%)	
Ex-smoker	364 (10.0%)		236 (6.5%)	
Yes	1295 (11.4%)		977 (8.6%)	
Alcohol drinking		<0.001		<0.001
No	1035 (6.0%)		754 (4.4%)	
Yes	3170 (9.1%)		2555 (7.3%)	
Betel nut chewing		<0.001		<0.001
No	3033 (7.4%)		2419 (5.9%)	
Yes	874 (13.2%)		714 (10.8%)	

**Table 2:** Job characteristics by self-reported and self-reports of physician-diagnosed PUD.

Job Characteristics	Self-Report PUD *n* = 4205	*p*	Self-Reports of Physician-Diagnosed PUD *n* = 3309	*p*
Group		<0.001		<0.001
Firefighters	666 (7.1%)		515 (5.5%)	
Policemen	3539 (8.3%)		2794 (6.5%)	
Work duration (years)		<0.001		<0.001
<5	245 (3.2%)		168 (2.2%)	
5–19	967 (5.9%)		733 (4.5%)	
20–29	2603 (10.7%)		2065 (8.5%)	
>30	337 (11.3%)		332 (10.0%)	
Level of employing government		0.452		0.936
Central	638 (8.2%)		504 (6.5%)	
Local	3012 (7.9%)		2446 (6.4%)	
Manager		0.815		<0.001
Yes	947 (8.1%)		843 (7.2%)	
No	3205 (8.1%)		2441 (6.1%)	
On-call work		<0.001		<0.001
No	647 (7.2%)		611 (6.5%)	
At home ready	129 (9.6%)		78 (5.8%)	
In office ready	2865 (8.0%)		2216 (6.2%)	
Both	315 (10.6%)		244 (8.2%)	
Overnight shifts		0.152		0.078
No	319 (9.0%)		250 (7.1%)	
Yes	3059 (8.3%)		2320 (6.3%)	
Work schedule		<0.001		<0.001
Fixed	589 (7.6%)		556 (7.2%)	
Work one days and one free	174 (6.9%)		135 (5.4%)	
Work two days and one free	373(7.7%)		276 (5.7%)	
Work shift	1343 (7.5%)		1065 (5.9%)	
Non-fixed	667 (9.7%)		480 (7.0%)	

**Table 3:** Percentages of high burnout, high workload, high job support, and PUD among firefighters and policemen.

Variables	Firefighters *n* = 9328	Policemen *n* = 42798	*p*
High burnout	2257 (24.2%)	9630 (22.5%)	0.002
High workload	4216 (45.2%)	12,240 (28.6%)	<0.001
High job support	4449 (47.7%)	20,628 (48.2%)	0.454
PUD diagnosed by physician	515 (5.5%)	2794 (6.5%)	<0.001
Self-report PUD by themselves	666 (7.1%)	3539 (8.3%)	<0.001

**Table 4:** PUD reported by participants and self-reports of physician-diagnosed affected by burnout, workload, and job support, using multiple logistic regression adjusted for groups, educational level, marital status, duration of job, levels of employing government, manager, smoking, alcohol, and betel nut chewing.

Model	Self-Report PUD	Self-Reports of Physician-Diagnosed PUD
Crude model	cOR (95%CI)	cOR (95%CI)
Burnout		
Low	1	1
High	1.40 ** (1.28–1.54)	1.77 ** (1.63–1.93)
Workload		
Low	1	1
High	1.30 ** (1.19–1.41)	1.34 ** (1.24–1.45)
Job support		
Low	1	1
High	0.68 ** (0.62–0.74)	0.56 ** (0.52–0.61)
Adjusted model	aOR (95%CI)	aORs (95%CI)
Burnout		
Low	1	1
High	1.54 ** (1.39–1.72)	1.92 ** (1.74–2.11)
Workload		
Low	1	1
High	1.42 ** (1.29–1.57)	1.55 ** (1.41–1.70)
Job support		
Low	1	1
High	0.73 ** (0.66–0.80)	0.60 ** (0.55–0.66)

Note. ** *p* < 0.01.

**Table 5:** Interaction between burnout and job support on the prevalence of peptic ulcer diseases (PUD) reported by participants and self-reported diagnosed by physicians using multiple logistic regression adjusted for groups, educational level, marital status, duration of job, levels of employing government, position of superior, alcohol drinking, smoking and betel nut chewing.

Model		Self-Report PUD		Self-Reports of Physician-Diagnosed	
**Crude model**		cOR (95% CI)	*p*	cOR (95% CI)	*p*
Burnout	Job support				
Low	Low	1		1	
Low	High	0.63 (0.58–0.68)	<0.001	0.74 (0.68–0.81)	<0.001
High	Low	1.64 (1.50–1.79)	<0.001	1.34 (1.21–1.48)	<0.001
High	High	1.04 (0.90–1.19)	0.557	0.87 (0.72–1.04)	0.131
**Adjusted model**		aOR (95% CI)	*p*	aOR (95% CI)	*p*
Burnout	Job support				
Low	Low	1		1	
Low	High	0.67 (0.61–0.75)	<0.001	0.78 (0.70–0.87)	<0.001
High	Low	1.78 (1.59–1.99)	<0.001	1.53 (1.35–1.73)	<0.001
High	High	1.34 (1.12–1.60)	0.010	1.07 (0.88v1.31)	0.383
